# Secretory Phospholipase A2 as a Promising Biomarker for Predicting Acute Chest Syndrome in Children With Sickle Cell Disease: A Systematic Review and Meta-Analysis

**DOI:** 10.7759/cureus.69053

**Published:** 2024-09-10

**Authors:** Mohammed Alsabri, Ahmed B Elsnhory, Omar Alattar, Noha Ahmed, Sarah Ashraf, Riffa Alassri

**Affiliations:** 1 Paediatrics, Brookdale University Hospital Medical Center, Brooklyn, USA; 2 General Practice, Al-Azhar University, Cairo, EGY; 3 Faculty of Medicine, Damascus University, Damascus, SYR; 4 Faculty of Medicine, Al-Azhar University, Cairo, EGY; 5 Faculty of Medicine, Menoufia University, Al Minufiyah, EGY; 6 Faculty of Medicine, Hama University, Hama, SYR

**Keywords:** acs, acute chest syndrome, biomarker, phospholipase a2, sickle cell disease

## Abstract

Acute chest syndrome (ACS) is a severe and potentially life-threatening complication of sickle cell disease (SCD). Early identification of patients at risk for ACS is crucial for timely intervention. There is a potential association between ACS and elevated levels of secretory phospholipase A2 (sPLA2), an enzyme involved in the breakdown of phospholipids. sPLA2 has emerged as a promising biomarker for predicting ACS. This systematic review and meta-analysis aimed to assess the diagnostic value of PLA2 in predicting ACS among children with SCD.

A comprehensive search was conducted across multiple databases, including MEDLINE, Embase, Cochrane Library, PubMed, and Web of Science. Studies assessing the relationship between sPLA2 levels and ACS in SCD patients were included. Pooled sensitivity, specificity, likelihood ratios, and the area under the receiver operating characteristic curve (AUC) were calculated to assess sPLA2's diagnostic accuracy.

There is a potential association between significant association between elevated sPLA2 levels and increased ACS risk in SCD patients. The pooled sensitivity of sPLA2 for predicting ACS was 0.766 (95% CI: 0.620-0.877), with a pooled specificity of 0.736 (95% CI: 0.680-0.787). The AUC of the summary receiver operating characteristic (SROC) curve was 0.84, indicating good discriminatory ability.

sPLA2 emerges as a promising biomarker for predicting ACS in SCD patients, potentially guiding risk stratification and early intervention strategies to enhance patient outcomes. Nonetheless, further prospective studies are warranted to validate its clinical utility and standardize sPLA2 assay protocols.

## Introduction and background

Acute chest syndrome (ACS) is the primary cause of mortality in individuals with sickle cell disease (SCD) and is responsible for the second-highest number of hospitalizations [1]. According to recent reports, severe ACS cases often exhibit bone marrow fat embolism. The release of free fatty acids, caused by the inflammatory mediator secretory phospholipase A2 (sPLA2), is believed to be responsible for the acute lung injury associated with fat embolism syndrome [2]. Several biomarkers have been investigated as potential predictors of ACS in SCD patients, including C-reactive protein (CRP), interleukin-6 (IL-6), and sPLA2 [2-3]. sPLA2 is an enzyme that breaks down phospholipids, creating lysophospholipids and free fatty acids [4]. When arachidonic acid is produced as the fatty acid, several inflammatory mediators, such as thromboxanes, leukotrienes, and prostaglandins, are generated. These mediators, along with free fatty acids, have been linked to acute lung injury [2]. Identifying patients with ACS is critical for initiating prompt and appropriate treatment, which can significantly improve patient outcomes. A systematic review of the value of sPLA2 as a predictor of ACS compared to other biomarkers in SCD patients is important because the information that will be obtained can assist clinicians in making knowledgeable choices regarding the utilization of sPLA2 in diagnosing and treating ACS In SCD patients presenting with chest pain. If sPLA2 is found to be a better predictor of ACS than other biomarkers, it could become a valuable tool in the early identification of ACS, leading to earlier initiation of appropriate treatment and improved patient outcomes [2]. In conclusion, a systematic review of the literature on the value of sPLA2 as a predictor of ACS in SCD patients presenting with chest pain to the emergency department is necessary to provide clinicians and researchers with a better understanding of the diagnostic and prognostic value of sPLA2 in ACS. The results of this review can inform clinical decision-making and guide future research on the use of sPLA2 as a biomarker for ACS in SCD patients. Enhancing the recognition and treatment of ACS can ultimately aid in decreasing the morbidity and mortality rates linked with this severe complication of SCD [5].

## Review

Materials and methods

Review Protocol and Guidelines

This review was conducted per Preferred Reporting Items for Systematic Reviews and Meta-Analyses (PRISMA) guidelines [6]. A literature search was conducted using the following databases: MEDLINE, Embase, Cochrane Library, Web of Science, and Scopus. Articles were screened using the Covidence (Alfred Health, Melbourne, Australia)systematic review tool by two independent reviewers to assess the quality, inclusion, and exclusion of the literature in the study. Covidence is a systematic review production tool for title/abstract screening, full-text screening, data abstraction, and quality assessment [7]. Based on the PRISMA guidelines, the investigator (M.A.) created the review protocol and a search strategy. Our research question was developed following the key elements of the PICO framework: Participants, Interventions, Comparison, and Outcomes [8]. The protocol was registered in the International Prospective Register of Systematic Reviews (PROSPERO) 2023 (CRD42023430266) and is included in the supplementary information. 

Inclusion and Exclusion Criteria

We sought both qualitative and quantitative primary studies investigating the accuracy of sPLA2 as a predictor and diagnostic biomarker for identifying ACS in patients with vaso-occlusive crises (VOC). Inclusion criteria were defined as follows:

1 - Peer-reviewed interventional or observational studies,

2 - Published in English,

3 - Involving pediatric patients (<21 years old) with SCD.

The exclusion criteria were as follows:

1 - Secondary or tertiary articles,

2 - Non-English publications,

3 - Studies involving adult populations (>21 years old), except where pediatric data constituted a significant part of the study.

Data Sources

The literature search strategies were developed using medical subject headings (MeSH) and text words related to ACS and biomarkers. The following databases were queried for identifying the peer-reviewed literature: MEDLINE, Cochrane Library, Web of Science, Scopus, and Embase. To ensure literature saturation, we scanned the reference lists of included studies and relevant reviews identified through the screening. Finally, we circulated a bibliography of the included articles to the systematic review team. The most recent search was conducted in July 2023.

Study Selection

Screening was completed in two stages using the Covidence systematic review management program [7]. Articles were screened for relevance based on the title and abstract and then evaluated for inclusion based on the full text. Two reviewers (A.B. and O.A.) independently screened the titles and abstracts. The selection was focused only on peer-reviewed published studies. The reviewers read the full-text articles obtained and selected those that met all inclusion criteria. A third author (M.A.) assisted in resolving any disagreements through consensus agreement. The details of the screening and selection process were documented in a PRISMA flow diagram presented in the final review, as seen below [9].

Quality Assessment of Studies

Two assessors (A.B. and O.A.) independently rated the quality of the studies using the Newcastle-Ottawa Scale (NOS) assessment [10]. In addition, if discrepancies were presented, these were resolved through discussion and consensus between the analysts.

The quality assessment of the included trials was conducted utilizing the Cochrane risk of bias assessment tool 1 (RoB-1), specifically designed for interventional studies [11]. This assessment tool comprised multiple parameters, including selection bias, performance bias, detection bias, attrition bias, reporting bias, and other potential sources of bias. Each trial underwent a risk of bias evaluation, with the authors categorizing the level of bias as "high," "low," or "unclear" for each parameter assessed. To ensure accuracy and consistency, any discrepancies during the evaluation process were resolved through discussions between the investigators or involving a third assessor (M.A.).

Data Extraction

Data extraction was performed using a standardized, offline data extraction sheet. The extracted data included the following: first author's name, study type, participant characteristics, SCD genotypes, the timing of sample collection, and outcomes recorded.

Statistical Analysis

The statistical analysis using Comprehensive Meta-Analysis (CMA) software (Biostat, Englewood, NJ) assessed the diagnostic performance of the test by analyzing sensitivity, specificity, positive likelihood ratio (+LR), negative likelihood ratio (-LR), and summary receiver operating characteristic (SROC) curve measures. Sensitivity and specificity were evaluated to determine the test's ability to identify individuals with and without the condition of interest correctly. Likelihood ratios (+LR and -LR) were calculated to assess the impact of positive and negative test results on the odds of having the condition. The SROC curve was used to visualize the trade-off between sensitivity and specificity, with the area under the curve (AUC) providing an overall measure of diagnostic accuracy. The homogeneity of the data was evaluated by assessing the I2 statistic using the CMA software. When the I2 statistic was less than 50%, it indicated that the data were considered homogeneous. 

Results

Literature Search and Study Selection

**Figure 1:**
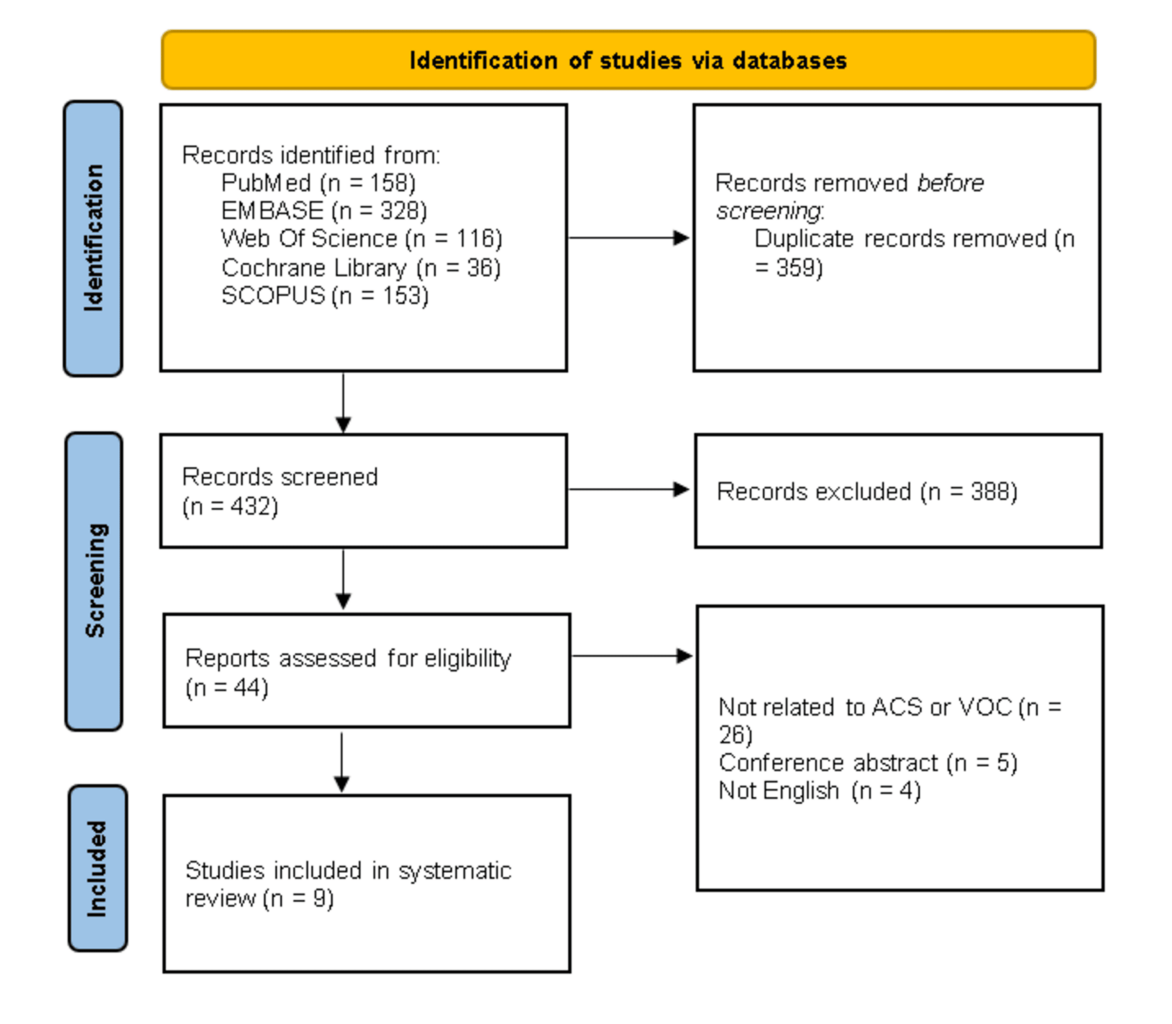
PRISMA flow diagram. The PRISMA diagram outlines our search and selection process for this systematic review. PRISMA, Preferred Reporting Items for Systematic Reviews and Meta-Analyses

A systematic search of multiple databases, including PubMed, Embase, Web of Science, Cochrane Library, and SCOPUS, yielded a total of 791 records. After removing duplicates, 432 records underwent title and abstract screening, leading to the exclusion of 388 studies. Subsequently, 44 studies were screened for eligibility, out of which nine studies met the inclusion criteria (Figure 1) [2,11-18].

Characteristics of Included Studies

The nine studies included in this review varied in study design, sample size, and methodology. These studies collectively explored the association between sPLA2 levels and ACS in pediatric SCD patients. However, only three studies were used to assess the diagnostic performance metrics of sPLA2 due to the availability of relevant data. Of the nine studies included, eight were conducted in the USA and one in the UK. It should be noted that although the UK study provided valuable insights, it was not included in the diagnostic performance analysis due to the lack of necessary data (Table 1).

**Table 1:** The summary of characteristics of the selected studies ACS, acute chest syndrome; HbSC, hemoglobin sickle C disease; HbSS, sickle cell anemia; PMN, polymorphonuclear neutrophil; SCD, sickle cell disease; sPLA2, secretory phospholipase A2; VOC, vaso-occlusive crises

Study ID	Country	Study design	Participant characteristics	Sickle cell disease genotypes	Type of PLA2 measured	Timing of sample collection	Outcomes
Styles et al. 1996 [2]	USA	Case-control study	65 (35 SCD patients, 11 pneumonia patients, 19 normal control). Age: range 1-20 years, mean = 11 years.	NA	sPLA	Upon admission and during the hospitalizations.	Elevating of sPLA2 levels.
Styles et al. 2000 [18]	USA	Prospective cohort study	14 SCD patients during 21 hospital admissions for VOC Age: range 1.5-20 years, mean = 12.6 ± 4.9 years.	NA	sPLA2	Daily during hospitalization.	The presence of ACS.
Styles et al. 2006 [15]	USA	Randomized controlled trials	14 SCD patients with 15 VOC (one patient was enrolled twice). Age: mean = 15 ± 4 years.	NA	sPLA2	Daily during hospitalization.	Preventing ACS.
Styles et al. 2012 [19]	USA	Randomized controlled trials + prospective cohort study	Component 1: 10 randomized SCD patients. Component 2: 203 SCD patients (96 adults, 107 children).	HbSS, SC or S-β0 or S-β+ thalassaemia	sPLA2	A maximum of three daily SPLA2 levels were determined.	(1) The presence of ACS; (2) preventing ACS.
Ballas et al. 2006 [13]	USA	Prospective cohort study	43 SCD with ACS (18 male, 23 female). Age: mean = 15.4 ± 10.3 years.	NA	sPLA2	At baseline (time of diagnosis) and serially thereafter up to day 22-35 follow-up visits.	Changes in sPLA2 values over days.
Bargom et al. 2005 [14]	USA	Prospective cohort study	20 hospitalized SCD patients (13 with VOC and seven with ACS).	NA	sPLA2	Between five and 10 serum samples were collected during the course of hospitalization.	Change in CRP values over days.
Naprawa et al. 2005 [17]	USA	Prospective cohort study	51 SCD patients (32 males, 19 females). Age: IQR = 6.9-16.6 years, median age = 12.1 years.	HbSS, SC or S-β0 or S-β+ thalassaemia	sPLA2	Samples were collected in the ED except for one obtained in the outpatient hematology clinic.	The presence of ACS.
Mollapour et al 1997 [16]	UK	In-vitro study	94 (32 SCD patients, 62 non-SCD patients)	HbSS	PLA2	NA	Neutrophil activation.
Ball et al. 2012 [12]	USA	In-vitro study + prospective cohort study	28 SCD patients (13 with VOC and 15 with ACS) (15 males, 13 females). Age: mean for VOC = 11.6 ± 1.3 year, mean for ACS = 8.4 ± 1.3 years.	HbSS or HbSC disease	sPLA2	During the annual comprehensive clinic visit or on day 1 of admission for VOC or ACS, before transfusion.	(1) The presence of ACS. (2) PMN-mediated endothelial cytotoxicity.

Diagnostic Performance Analysis

**Figure 2:**
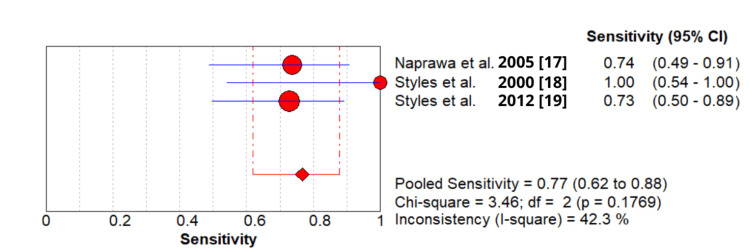
Sensitivity of secretory phospholipase A2 (sPLA2) test in patients with acute chest syndrome based on included studies.

The diagnostic performance analysis, based on the three selected studies, revealed the following:

1 - Pooled sensitivity: 0.766 (95% CI: 0.620-0.877) (Figure 2).

**Figure 3:**
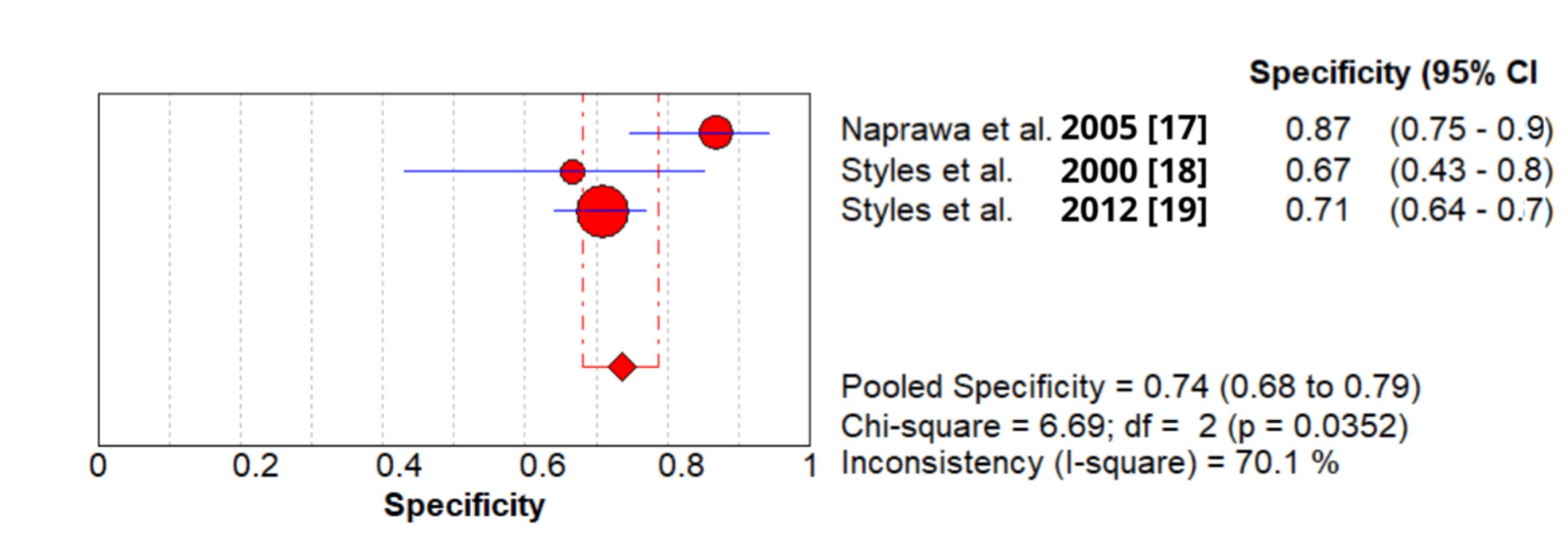
Specificity of secretory phospholipase A2 (sPLA2) test in patients with acute chest syndrome based on included studies.

2 - Pooled specificity: 0.736 (95% CI: 0.680-0.787) (Figure 3).

**Figure 4:**
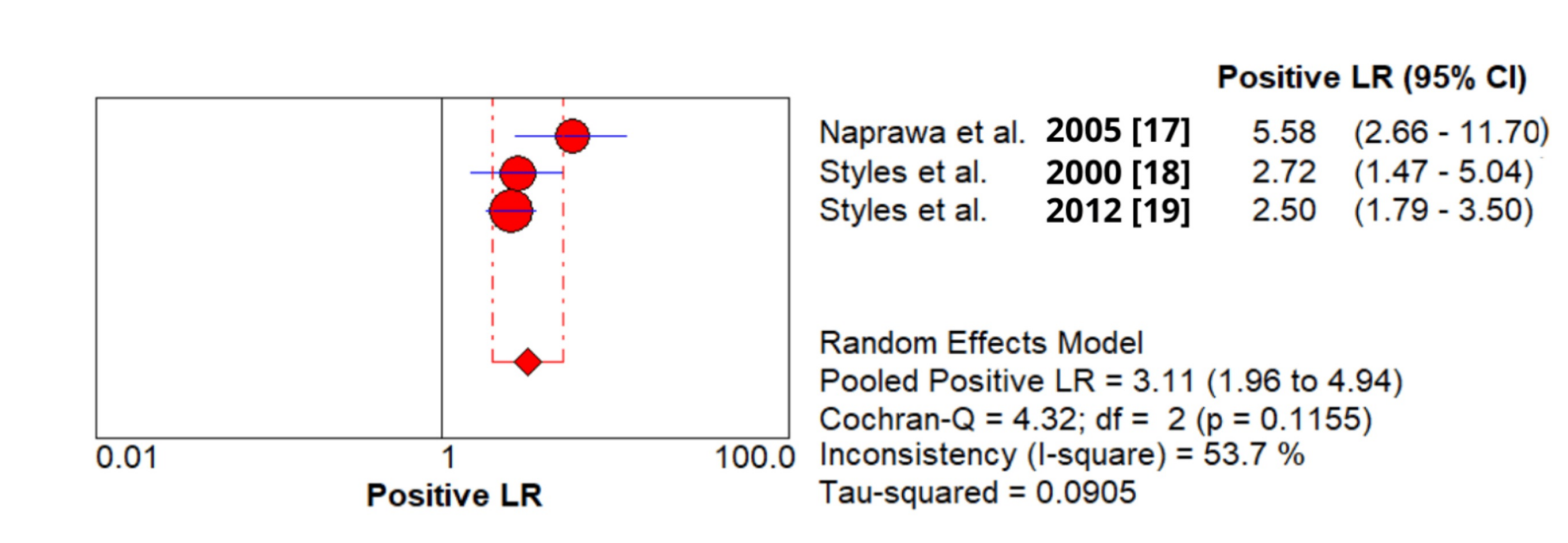
The pooled analysis of the positive likelihood ratio of secretory phospholipase A2 (sPLA2) test in patients with acute chest syndrome based on included studies.

3 - Pooled positive likelihood ratio (+LR): 3.108 (95% CI: 1.956-4.937) (Figure 4).

**Figure 5:**
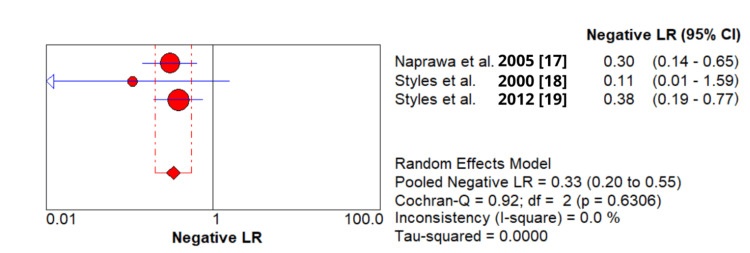
The pooled analysis of the negative likelihood ratio of secretory phospholipase A2 (sPLA2) test in patients with acute chest syndrome based on included studies.

4 - Pooled negative likelihood ratio (-LR): 0.332 (95% CI: 0.201-0.548) (Figure 5).

**Figure 6:**
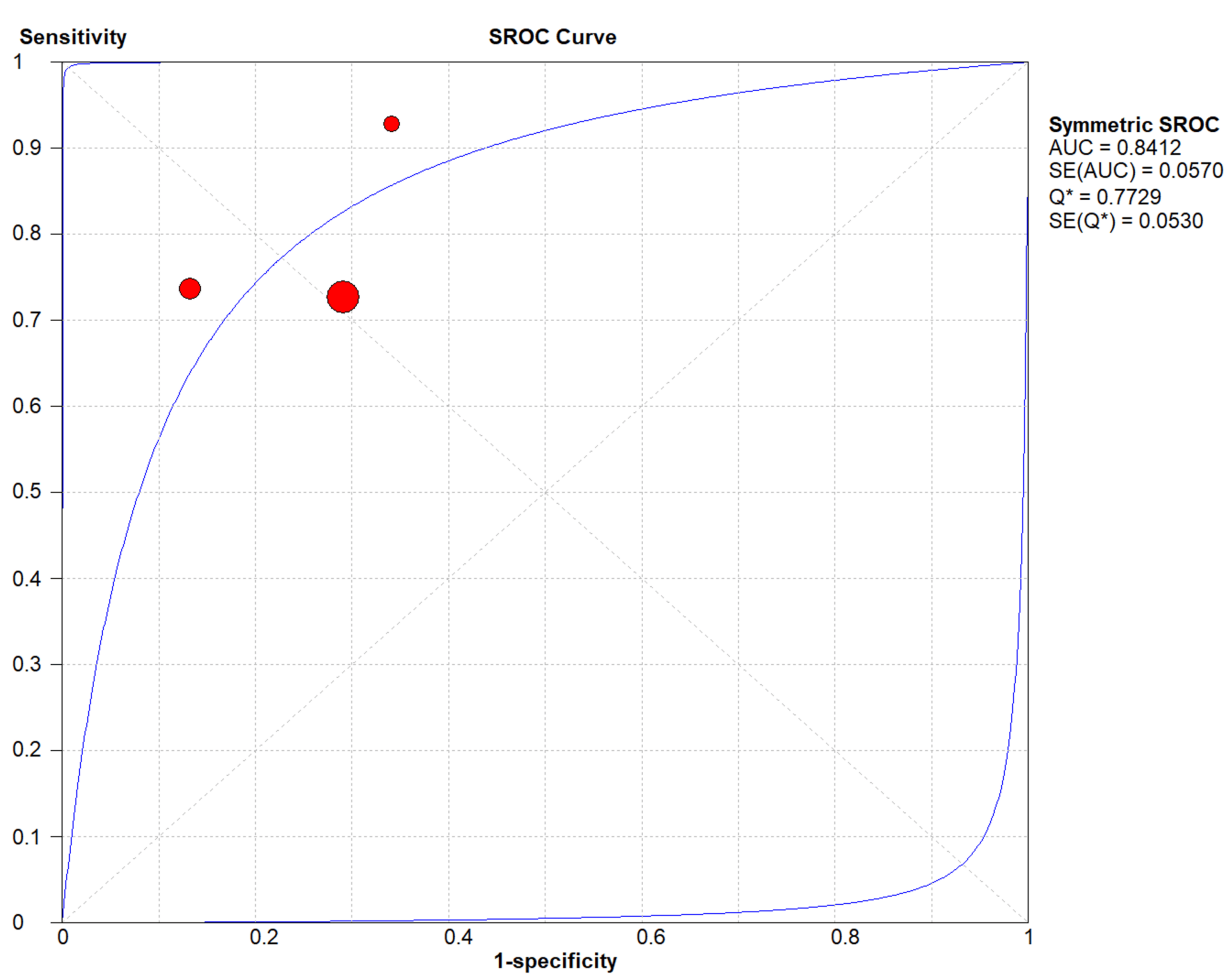
The symmetric summary receiver operating characteristic of secretory phospholipase A2 (sPLA2) test in patients with acute chest syndrome based on included studies. [17-19]

5 - AUC: 0.84 (standard error: 0.057) (Figure 6).

These results indicate that sPLA2 demonstrates a moderate ability to identify ACS in SCD patients, with the AUC suggesting reasonably good discriminatory ability.

Risk of Bias Assessment

Most of our included studies showed good quality on the NOS assessment. However, just one study did not have an adequate follow-up period. Table 2 shows the detailed assessment of each study and their score in the individual items of the three major domains of NOS assessment (selection, comparability, and outcome). For randomized controlled trial (RCT) studies, we used the Cochrane tool RoB-1 to assess them, and it showed poor quality due to insufficient information about sequence generation and allocation concealment (Table 3).

**Table 2:** The Newcastle-Ottawa Scale tool for risk of bias assessment of the selected studies For each criterion assessed using the Newcastle-Ottawa Scale, a notation of “*” is used to indicate that the study successfully meets the specified criteria.

Authors	Selection				Comparability of cohorts	Outcome			Total score
Prospective cohort studies	Representativeness of the exposed cohort	Selection of the non-exposed cohort	Ascertainment of exposure	The outcome of interest not present at the study start		Assessment	Length of follow-up	Adequacy of follow-up of cohorts	
Styles et al. 2000 [18]	*	*	*	*	*	*	*	*	8
Styles et al. 2012 [19]	*	*	*	*	*	*	*	*	8
Ballas et al. 2006 [13]	*	*	*	*	*	*	*	*	8
Bargoma et al. 2005 [14]	*	*	*	*	*	*	*	*	8
Naprawa et al. 2005 [17]	*	*	*	*	*	*	*	*	8
Ball et al. 2012 [12]	*	*	*	*	*	*			6
Case-control studies	Is the case definition adequate?	Representativeness of the cases	Selection of controls	Definition of controls	Comparability of cohorts	Ascertainment of exposure	The same method of ascertainment for cases and controls	Non-response rate	Total score
Styles et al. 1996 [2]	*	*		*	*	*	*	*	7

**Table 3:** Risk of bias 1 tool for assessment of included studies ACS, acute chest syndrome; CXR, chest X-ray

Study ID	The Cochrane Collaboration’s tool for assessing the risk of bias													
	Random sequence generation (selection bias)		Allocation concealment (selection bias)		Blinding of participants and personnel (performance bias)		Blinding of outcome assessment (detection bias)		Incomplete outcome data (attrition bias)		Selective reporting (reporting bias)		Other bias	
	Low/high/unclear risk of bias	Reason	Low/high/unclear risk of bias	Reason	Low/high/unclear risk of bias	Reason	Low/high/unclear risk of bias	Reason	Low/high/unclear risk of bias	Reason	Low/high/unclear risk of bias	Reason	Low/high/unclear risk of bias	Reason
Styles et al. 2006 [15]	Unclear risk of bias	"Patients were randomized to receive either standard care or a single packed red blood cell transfusion of approximately 10 cc/kg."	Unclear risk of bias	"Patients were randomized to receive either standard care or a single packed red blood cell transfusion of approximately 10 cc/kg."	High risk of bias	"Due to the nature of the intervention (blood transfusion), it was impossible to blind either the patient or the medical staff as to the study assignment."	Low risk of bias	"The determination of whether a patient had developed a new pulmonary infiltrate was made by a radiologist who was blinded to the study."	Low risk of bias	"In five of these 22 events, the family refused enrolment because of a fear of transfusion. Outcome data on these five patients were subsequently reviewed to ensure that the enrolled population was representative of all eligible patients."	Low risk of bias	The published study reported its expected primary outcome.	None	None
Styles et al. 2012 [19]	Unclear risk of bias	Insufficient information	Unclear risk of bias	Insufficient information	High risk of bias	"Transfusion therapy cannot be safely blinded compounds these potential issues."	Low risk of bias	"If a CXR was reported positive but a diagnosis of ACS was not made, these CXR reports were retrieved from the sites and blindly and independently reviewed by Drs. Styles and Miller to determine whether or not ACS was present."	Low risk of bias	No missing data	Low risk of bias	The published study reported its expected primary outcomes.	None	None

Discussion

This review represents the first comprehensive meta-analysis to evaluate the potential of sPLA2 as a diagnostic tool for ACS in pediatric patients with SCD presenting with or admitted with VOC. Our systematic approach across multiple databases ensured the inclusion of relevant studies, with clear inclusion criteria to strengthen the validity of our findings [2,17-18].

The analysis focused on three studies that provided sufficient data to evaluate the diagnostic performance of sPLA2. These studies consistently demonstrated a significant positive correlation between elevated sPLA2 levels and the incidence of ACS in pediatric SCD patients [17-19]. Notably, sPLA2 levels were found to rise before the onset of ACS, indicating its potential as an early diagnostic marker [17,19]. This early rise in sPLA2 suggests that it could be used in clinical practice to identify at-risk patients before the full development of ACS, potentially allowing for earlier interventions and improved outcomes.

However, it is crucial to note that this review did not compare sPLA2 with other biomarkers, nor did it assess the role of sPLA2 in diagnosing VOC, as all patients included in the studies were already diagnosed with VOC. The focus on ACS diagnosis within the context of VOC is critical, given that VOC is a common complication in SCD and frequently precedes the onset of ACS [2]. The studies included in the analysis did not provide data on the prognostic value of sPLA2, as there was no follow-up on outcomes related to elevated sPLA2 levels. Therefore, our conclusions are limited to the diagnostic utility of sPLA2 in the context of ACS in pediatric SCD patients [17-19]. Further research should explore whether sPLA2 can also predict the severity of ACS or other long-term outcomes, which would enhance its clinical utility.

The exclusion of adult studies limits the generalizability of our findings. While sPLA2 has shown promise in pediatric populations, its diagnostic accuracy in adults with SCD remains unclear and warrants further investigation [19]. The pathophysiology of ACS may differ between pediatric and adult populations, potentially influencing the performance of biomarkers like sPLA2 [13]. Therefore, the current findings should be interpreted with caution when considering their applicability to adult patients. Future studies should include a broader age range to determine if the diagnostic value of sPLA2 is consistent across different age groups.

Additionally, the limited number of included studies and the methodological variations among them highlight the need for larger, more standardized studies to validate the use of sPLA2 as a diagnostic marker for ACS. The variation in study design, sample size, and patient populations across the included studies may have introduced heterogeneity into our analysis, which could affect the reliability of our findings [10,11]. To address these limitations, future research should focus on standardizing the measurement of sPLA2 levels, including consistent timing of sample collection and the use of uniform diagnostic criteria for ACS. Such standardization would allow for more accurate comparisons across studies and enhance the robustness of the conclusions drawn.

Given the potential clinical implications, sPLA2 could enhance risk stratification in pediatric SCD patients by facilitating early identification of those at risk of developing ACS. This biomarker could be particularly useful in settings where clinical resources are limited and rapid, reliable diagnostic tools are needed [14]. Combining sPLA2 with other biomarkers might further improve the accuracy of ACS prediction, providing a more comprehensive assessment of a patient’s risk and guiding more personalized treatment strategies. For instance, the integration of sPLA2 with inflammatory markers like CRP or IL-6 could help differentiate between ACS and other complications of SCD, thereby improving clinical decision-making [2,12].

Despite the promising findings, our review also underscores the importance of expanding research efforts to include direct comparisons between sPLA2 and other biomarkers used in the diagnosis of ACS. Such comparative studies could clarify whether sPLA2 offers any significant advantages over existing markers or if it should be used in conjunction with them [14]. Furthermore, studies that evaluate the cost-effectiveness of sPLA2 as a diagnostic tool are needed to justify its implementation in clinical practice, particularly in resource-constrained settings [14].

Lastly, the potential impact of interventions such as blood transfusions, which have been shown to reduce sPLA2 levels and improve clinical outcomes in ACS, warrants further exploration. Understanding how treatment modalities influence sPLA2 levels could provide insights into its role not just as a diagnostic marker but also as a tool for monitoring treatment efficacy [15].

## Conclusions

In conclusion, this systematic review highlights the potential of sPLA2 as an early diagnostic marker for ACS in pediatric SCD patients. The consistent correlation between elevated sPLA2 levels and the onset of ACS across the included studies suggests that sPLA2 could facilitate timely intervention and improve clinical outcomes. However, larger studies, including direct comparisons with other biomarkers, are needed to further validate its clinical utility and cost-effectiveness.
